# Complete Genome Sequence of *Methanobacterium electrotrophus* Strain YSL, Isolated from Coastal Riverine Sediments

**DOI:** 10.1128/MRA.00752-21

**Published:** 2021-09-30

**Authors:** Shiling Zheng, Fanghua Liu

**Affiliations:** a CAS Key Laboratory of Coastal Environmental Processes and Ecological Remediation, Yantai Institute of Coastal Zone Research, Chinese Academy of Sciences, Yantai, People’s Republic of China; b National-Regional Joint Engineering Research Center for Soil Pollution Control and Remediation in South China, Guangdong Key Laboratory of Integrated Agro-environmental Pollution Control and Management, Institute of Eco-environmental and Soil Sciences, Guangdong Academy of Sciences, Guangzhou, People’s Republic of China; c Laboratory for Marine Biology and Biotechnology, Pilot National Laboratory for Marine Science and Technology, Qingdao, People’s Republic of China; Montana State University

## Abstract

Methanobacterium electrotrophus strain YSL was isolated from enriched microbial aggregates from a coastal riverine sediment sample from Shandong Province, China. The genome of YSL was sequenced with the PacBio Sequel platform and contained three plasmids in addition to the chromosome. A total of 2,521 protein-coding genes and 58 RNA genes were predicted.

## ANNOUNCEMENT

*Methanobacterium*, the genus of hydrogenotrophic methanogens, is widely distributed in coastal riverine sediments (1–3). We previously reported a strain of *Methanobacterium*, designated Methanobacterium electrotrophus strain YSL (*Methanobacterium* sp. strain YSL), which was not able to use H_2_ as an electron donor for methane production but was capable of directly accepting electrons from Geobacter metallireducens for the reduction of carbon dioxide to methane in defined cocultures (4).

M. electrotrophus strain YSL was isolated from enriched microbial aggregates (1) that had been serially diluted in mineral salts (MS) medium. The Hungate roll-tube technique was then performed several times to isolate a single colony, which was further purified with the streak plate method. A single colony was cultured in MS liquid medium with sodium formate (40 mmol liter^−1^) as the electron donor (4). Incubations were at 30°C.

Genomic DNA of M. electrotrophus strain YSL was extracted according to the manufacturer’s instructions for a bacterial DNA extraction minikit (Onrew, China). The quality and concentrations of DNA extracts were determined by standard gel electrophoresis and analysis with a Qubit fluorometer (Invitrogen, USA). The qualified genomic DNA was fragmented with a 26-gauge needle, and fragments larger than 20 kb were selected using the BluePippin system. After end repair, A-tailing and sequencing adapter ligation of fragments were performed, and a qualified DNA library was prepared for complete genome sequencing with the PacBio Sequel single-molecule real-time (SMRT) sequencing technology (Pacific Biosciences).

After quality filtering of raw data using MECAT2 (5) with default settings (default parameters were used for all software unless otherwise noted), 62,441 reads, with an *N*_50_ value of 12,977 bp, were assembled using SMRT Link v5.1.0 (https://www.pacb.com/support/software-downloads) to generate 4 contigs, with a maximum contig length of 2,509,437 bp (Table 1). Assembly polishing was performed using Arrow software (https://github.com/PacificBiosciences/GenomicConsensus). Lastly, Bandage (6) visualization was used to analyze the genome assemblies, and a circular chromosome and three plasmids were obtained (Table 1). In total, 2,521 protein-coding genes and 58 RNA genes, including 49 tRNAs, 7 rRNAs (three 5S rRNAs, two 16S rRNAs, and two 23S rRNAs), and 2 noncoding RNAs (ncRNAs), were annotated using the best-placed reference protein set and the GeneMarkS-2+ annotation method in the NCBI Prokaryotic Genome Annotation Pipeline (PGAP) (7) and were deposited in the GenBank database. In addition, 261 putative virulence factors in the genome were annotated by using the Virulence Factors Database (VFDB) (8).

**TABLE 1:** Genome characteristics of *Methanobacterium electrotrophus* strain YSL

Contig	Type	Length (bp)	GC content (%)	Circular^*a*^	GenBank accession no.
1	Chromosome	2,509,437	39.36	T	JAHQCO010000001
14	Plasmid	108,588	38.95	T	JAHQCO010000002
20	Plasmid	85,704	37.45	F	JAHQCO010000003
45	Plasmid	6,921	36.18	F	JAHQCO010000004

a Circular sequence; T, true; F, false.

### Data availability.

The M. electrotrophus strain YSL whole-genome sequencing project has been deposited in DDBJ/ENA/GenBank under the accession number JAHQCO000000000 and under BioProject accession number PRJNA733324 and BioSample accession number SAMN19375714. The version described in this paper is version JAHQCO010000000 and consists of sequences JAHQCO010000001 to JAHQCO010000004. The accession number for raw sequences is SRR15021169.
